# Supplementary material for: Referrals for physical therapy for osteoarthritis during the COVID-19 pandemic: A retrospective analysis
Source: PLoS One. 2021 Nov 5;16(11):e0259679. doi: 10.1371/journal.pone.0259679 (PMC8570525; doi:10.1371/journal.pone.0259679)
Supplement: S1 File — 206 applicable ICD-10 diagnosis codes for OA (see Supplemental Data 1) that were linked to PT referrals in our data set. (DOCX) [file pone.0259679.s001.docx]

**OA ICD 10 Codes**

| M13.131 | Monoarthritis, not elsewhere classified, right wrist |
| --- | --- |
| M13.14 | Monoarthritis, not elsewhere classified, hand |
| M13.80 | Other specified arthritis, unspecified site |
| M13.81 | Other specified arthritis, shoulder |
| M13.819 | Other specified arthritis, unspecified shoulder |
| M13.832 | Other specified arthritis, left wrist |
| M13.842 | Other specified arthritis, left hand |
| M16.50 | Unilateral post-traumatic osteoarthritis, unspecified hip |
| M17.9 | Osteoarthritis of knee, unspecified |
| M18.0 | Bilateral primary osteoarthritis of first carpometacarpal joints |
| M18.3 | Unilateral post-traumatic osteoarthritis of first carpometacarpal joint |
| M18.9 | Osteoarthritis of first carpometacarpal joint, unspecified |
| M19 | Other and unspecified osteoarthritis |
| M19.04 | Primary osteoarthritis, hand |
| M19.079 | Primary osteoarthritis, unspecified ankle and foot |
| M19.12 | Post-traumatic osteoarthritis, elbow |
| M19.121 | Post-traumatic osteoarthritis, right elbow |
| M19.139 | Post-traumatic osteoarthritis, unspecified wrist |
| M19.179 | Post-traumatic osteoarthritis, unspecified ankle and foot |
| M19.212 | Secondary osteoarthritis, left shoulder |
| M19.241 | Secondary osteoarthritis, right hand |
| M19.279 | Secondary osteoarthritis, unspecified ankle and foot |
| M19.92 | Post-traumatic osteoarthritis, unspecified site |
| M13.111 | Monoarthritis, not elsewhere classified, right shoulder |
| M13.129 | Monoarthritis, not elsewhere classified, unspecified elbow |
| M13.132 | Monoarthritis, not elsewhere classified, left wrist |
| M13.84 | Other specified arthritis, hand |
| M13.85 | Other specified arthritis, hip |
| M15.3 | Secondary multiple arthritis |
| M15.9 | Polyosteoarthritis, unspecified |
| M13.112 | Monoarthritis, not elsewhere classified, left shoulder |
| M13.12 | Monoarthritis, not elsewhere classified, elbow |
| M13.13 | Monoarthritis, not elsewhere classified, wrist |
| M13.142 | Monoarthritis, not elsewhere classified, left hand |
| M13.17 | Monoarthritis, not elsewhere classified, ankle and foot |
| M13.172 | Monoarthritis, not elsewhere classified, left ankle and foot |
| M13.811 | Other specified arthritis, right shoulder |
| M13.829 | Other specified arthritis, unspecified elbow |
| M13.88 | Other specified arthritis, other site |
| M15 | Polyosteoarthritis |
| M16.30 | Unilateral osteoarthritis resulting from hip dysplasia, unspecified hip |
| M17.0 | Bilateral primary osteoarthritis of knee |
| M17.32 | Unilateral post-traumatic osteoarthritis, left knee |
| M18.1 | Unilateral primary osteoarthritis of first carpometacarpal joint |
| M18.31 | Unilateral post-traumatic osteoarthritis of first carpometacarpal joint, right hand |
| M19.031 | Primary osteoarthritis, right wrist |
| M19.039 | Primary osteoarthritis, unspecified wrist |
| M19.071 | Primary osteoarthritis, right ankle and foot |
| M19.11 | Post-traumatic osteoarthritis, shoulder |
| M19.221 | Secondary osteoarthritis, right elbow |
| M19.232 | Secondary osteoarthritis, left wrist |
| M19.242 | Secondary osteoarthritis, left hand |
| M77.20 | Periarthritis, unspecified wrist |
| M13 | Other arthritis |
| M13.1 | Monoarthritis, not elsewhere classified |
| M13.11 | Monoarthritis, not elsewhere classified, shoulder |
| M13.141 | Monoarthritis, not elsewhere classified, right hand |
| M13.151 | Monoarthritis, not elsewhere classified, right hip |
| M13.152 | Monoarthritis, not elsewhere classified, left hip |
| M13.161 | Monoarthritis, not elsewhere classified, right knee |
| M13.812 | Other specified arthritis, left shoulder |
| M15.4 | Erosive (osteo)arthritis |
| M16.0 | Bilateral primary osteoarthritis of hip |
| M16.12 | Unilateral primary osteoarthritis, left hip |
| M16.32 | Unilateral osteoarthritis resulting from hip dysplasia, left hip |
| M16.7 | Other unilateral secondary osteoarthritis of hip |
| M17.12 | Unilateral primary osteoarthritis, left knee |
| M17.4 | Other bilateral secondary osteoarthritis of knee |
| M18.4 | Other bilateral secondary osteoarthritis of first carpometacarpal joints |
| M19.0 | Primary osteoarthritis of other joints |
| M19.129 | Post-traumatic osteoarthritis, unspecified elbow |
| M19.142 | Post-traumatic osteoarthritis, left hand |
| M19.149 | Post-traumatic osteoarthritis, unspecified hand |
| M26.649 | Arthritis of unspecified temporomandibular joint |
| M16.11 | Unilateral primary osteoarthritis, right hip |
| M16.3 | Unilateral osteoarthritis resulting from hip dysplasia |
| M16.31 | Unilateral osteoarthritis resulting from hip dysplasia, right hip |
| M16.51 | Unilateral post-traumatic osteoarthritis, right hip |
| M16.6 | Other bilateral secondary osteoarthritis of hip |
| M17.31 | Unilateral post-traumatic osteoarthritis, right knee |
| M18.11 | Unilateral primary osteoarthritis of first carpometacarpal joint, right hand |
| M19.01 | Primary osteoarthritis, shoulder |
| M19.021 | Primary osteoarthritis, right elbow |
| M19.049 | Primary osteoarthritis, unspecified hand |
| M19.112 | Post-traumatic osteoarthritis, left shoulder |
| M19.141 | Post-traumatic osteoarthritis, right hand |
| M19.219 | Secondary osteoarthritis, unspecified shoulder |
| M19.222 | Secondary osteoarthritis, left elbow |
| M19.239 | Secondary osteoarthritis, unspecified wrist |
| M19.272 | Secondary osteoarthritis, left ankle and foot |
| M19.93 | Secondary osteoarthritis, unspecified site |
| M26.641 | Arthritis of right temporomandibular joint |
| M26.642 | Arthritis of left temporomandibular joint |
| M77.21 | Periarthritis, right wrist |
| M13.121 | Monoarthritis, not elsewhere classified, right elbow |
| M13.159 | Monoarthritis, not elsewhere classified, unspecified hip |
| M13.169 | Monoarthritis, not elsewhere classified, unspecified knee |
| M13.8 | Other specified arthritis |
| M13.82 | Other specified arthritis, elbow |
| M13.852 | Other specified arthritis, left hip |
| M13.859 | Other specified arthritis, unspecified hip |
| M13.862 | Other specified arthritis, left knee |
| M13.87 | Other specified arthritis, ankle and foot |
| M16.2 | Bilateral osteoarthritis resulting from hip dysplasia |
| M17.1 | Unilateral primary osteoarthritis of knee |
| M18.10 | Unilateral primary osteoarthritis of first carpometacarpal joint, unspecified hand |
| M18.32 | Unilateral post-traumatic osteoarthritis of first carpometacarpal joint, left hand |
| M18.50 | Other unilateral secondary osteoarthritis of first carpometacarpal joint, unspecified hand |
| M19.032 | Primary osteoarthritis, left wrist |
| M19.22 | Secondary osteoarthritis, elbow |
| M19.231 | Secondary osteoarthritis, right wrist |
| M19.29 | Secondary osteoarthritis, other specified site |
| M19.91 | Primary osteoarthritis, unspecified site |
| M08.81 | Other juvenile arthritis, shoulder |
| M13.149 | Monoarthritis, not elsewhere classified, unspecified hand |
| M13.162 | Monoarthritis, not elsewhere classified, left knee |
| M13.171 | Monoarthritis, not elsewhere classified, right ankle and foot |
| M13.83 | Other specified arthritis, wrist |
| M13.831 | Other specified arthritis, right wrist |
| M13.839 | Other specified arthritis, unspecified wrist |
| M13.879 | Other specified arthritis, unspecified ankle and foot |
| M15.0 | Primary generalized (osteo)arthritis |
| M16.52 | Unilateral post-traumatic osteoarthritis, left hip |
| M16.9 | Osteoarthritis of hip, unspecified |
| M17.10 | Unilateral primary osteoarthritis, unspecified knee |
| M17.11 | Unilateral primary osteoarthritis, right knee |
| M17.2 | Bilateral post-traumatic osteoarthritis of knee |
| M17.3 | Unilateral post-traumatic osteoarthritis of knee |
| M17.5 | Other unilateral secondary osteoarthritis of knee |
| M18.12 | Unilateral primary osteoarthritis of first carpometacarpal joint, left hand |
| M18.5 | Other unilateral secondary osteoarthritis of first carpometacarpal joint |
| M18.52 | Other unilateral secondary osteoarthritis of first carpometacarpal joint, left hand |
| M19.019 | Primary osteoarthritis, unspecified shoulder |
| M19.02 | Primary osteoarthritis, elbow |
| M19.07 | Primary osteoarthritis ankle and foot |
| M19.13 | Post-traumatic osteoarthritis, wrist |
| M19.132 | Post-traumatic osteoarthritis, left wrist |
| M19.21 | Secondary osteoarthritis, shoulder |
| M19.211 | Secondary osteoarthritis, right shoulder |
| M19.90 | Unspecified osteoarthritis, unspecified site |
| M08.80 | Other juvenile arthritis, unspecified site |
| M08.812 | Other juvenile arthritis, left shoulder |
| M08.832 | Other juvenile arthritis, left wrist |
| M08.862 | Other juvenile arthritis, left knee |
| M08.89 | Other juvenile arthritis, multiple sites |
| M08.952 | Juvenile arthritis, unspecified, left hip |
| M08.969 | Juvenile arthritis, unspecified, unspecified knee |
| M13.10 | Monoarthritis, not elsewhere classified, unspecified site |
| M13.179 | Monoarthritis, not elsewhere classified, unspecified ankle and foot |
| M13.821 | Other specified arthritis, right elbow |
| M13.822 | Other specified arthritis, left elbow |
| M13.841 | Other specified arthritis, right hand |
| M13.86 | Other specified arthritis, knee |
| M13.861 | Other specified arthritis, right knee |
| M13.869 | Other specified arthritis, unspecified knee |
| M13.872 | Other specified arthritis, left ankle and foot |
| M15.8 | Other polyosteoarthritis |
| M16.1 | Unilateral primary osteoarthritis of hip |
| M16.10 | Unilateral primary osteoarthritis, unspecified hip |
| M16.5 | Unilateral post-traumatic osteoarthritis of hip |
| M17.30 | Unilateral post-traumatic osteoarthritis, unspecified knee |
| M18.2 | Bilateral post-traumatic osteoarthritis of first carpometacarpal joints |
| M18.30 | Unilateral post-traumatic osteoarthritis of first carpometacarpal joint, unspecified hand |
| M18.51 | Other unilateral secondary osteoarthritis of first carpometacarpal joint, right hand |
| M19.011 | Primary osteoarthritis, right shoulder |
| M19.012 | Primary osteoarthritis, left shoulder |
| M19.029 | Primary osteoarthritis, unspecified elbow |
| M19.041 | Primary osteoarthritis, right hand |
| M19.1 | Post-traumatic osteoarthritis of other joints |
| M19.111 | Post-traumatic osteoarthritis, right shoulder |
| M19.171 | Post-traumatic osteoarthritis, right ankle and foot |
| M19.172 | Post-traumatic osteoarthritis, left ankle and foot |
| M19.19 | Post-traumatic osteoarthritis, other specified site |
| M19.24 | Secondary osteoarthritis, hand |
| M19.249 | Secondary osteoarthritis, unspecified hand |
| M19.27 | Secondary osteoarthritis, ankle and foot |
| M13.119 | Monoarthritis, not elsewhere classified, unspecified shoulder |
| M13.122 | Monoarthritis, not elsewhere classified, left elbow |
| M13.139 | Monoarthritis, not elsewhere classified, unspecified wrist |
| M13.15 | Monoarthritis, not elsewhere classified, hip |
| M13.16 | Monoarthritis, not elsewhere classified, knee |
| M13.849 | Other specified arthritis, unspecified hand |
| M13.851 | Other specified arthritis, right hip |
| M13.871 | Other specified arthritis, right ankle and foot |
| M13.89 | Other specified arthritis, multiple sites |
| M16 | Osteoarthritis of hip |
| M16.4 | Bilateral post-traumatic osteoarthritis of hip |
| M17 | Osteoarthritis of knee |
| M18 | Osteoarthritis of first carpometacarpal joint |
| M19.022 | Primary osteoarthritis, left elbow |
| M19.03 | Primary osteoarthritis, wrist |
| M19.042 | Primary osteoarthritis, left hand |
| M19.072 | Primary osteoarthritis, left ankle and foot |
| M19.09 | Primary osteoarthritis, other specified site |
| M19.119 | Post-traumatic osteoarthritis, unspecified shoulder |
| M19.122 | Post-traumatic osteoarthritis, left elbow |
| M19.131 | Post-traumatic osteoarthritis, right wrist |
| M19.14 | Post-traumatic osteoarthritis, hand |
| M19.17 | Post-traumatic osteoarthritis, ankle and foot |
| M19.2 | Secondary osteoarthritis of other joints |
| M19.229 | Secondary osteoarthritis, unspecified elbow |
| M19.23 | Secondary osteoarthritis, wrist |
| M19.271 | Secondary osteoarthritis, right ankle and foot |
| M19.9 | Osteoarthritis, unspecified site |
| M26.643 | Arthritis of bilateral temporomandibular joint |
| Z82.61 | Family history of arthritis |
