# Supplementary material for: Referrals for physical therapy for osteoarthritis during the COVID-19 pandemic: A retrospective analysis
Source: PLoS One. 2021 Nov 5;16(11):e0259679. doi: 10.1371/journal.pone.0259679 (PMC8570525; doi:10.1371/journal.pone.0259679)
Supplement: S3 File — Permission to Use Fig 3 from the Sacramento County who publishes the COVID cases dashboard online. (DOCX) [file pone.0259679.s003.docx]

DHS COVID19 <COVID19@saccounty.net>

Mon 9/13/2021 8:50 AM

Dr. Kaur,

You are welcome to use screen shot data from our dashboard.



**From:** DHS Director <DHS-Director@saccounty.net>
**Sent:** Monday, September 13, 2021 8:44 AM
**To:** Manmeet Kaur <mmkaur@ucdavis.edu>
**Cc:** DHS COVID19 <COVID19@saccounty.net>
**Subject:** RE: Question about using COVID 19 Dashboard Diagram in Article

Good morning Dr. Kaur,

I am looping in the Public Health Team at [COVID19@saccounty.net](mailto:COVID19@saccounty.net) to respond to your request.

Regards,

**Michelle Gorre**

Department of Health Services

916-875-2001

**From:** Manmeet Kaur <[mmkaur@ucdavis.edu](mailto:mmkaur@ucdavis.edu)>
**Sent:** Sunday, September 12, 2021 3:20 PM
**To:** DHS Director <[DHS-Director@saccounty.net](mailto:DHS-Director@saccounty.net)>
**Subject:** Question about using COVID 19 Dashboard Diagram in Article

| \| **EXTERNAL EMAIL:** If unknown sender, do not click links/attachments. \| \| --- \| |
| --- | --- |

Hi,

I am a UC Davis Internal Medicine resident and I work at the County Clinic as a primary care physcian. I am working on a research study which I hope to publish through PLOS One on how the COVID 19 pandemic affected Osteoarthritis referrals for physical therapy.

I would like to use a screenshot of the COVID 19 Sacramento Dashboard in my study to show local trends of COVID 19 cases during this time. Please find the diagram attached. I will be referencing the source. Would you be okay with me using that diagram?

Thank you,

Manmeet Kaur, MD

UC Davis Medical Center

Sacramento County Clinic
